# Room-temperature helimagnetism in FeGe thin films

**DOI:** 10.1038/s41598-017-00201-z

**Published:** 2017-03-09

**Authors:** S. L. Zhang, I. Stasinopoulos, T. Lancaster, F. Xiao, A. Bauer, F. Rucker, A. A. Baker, A. I. Figueroa, Z. Salman, F. L. Pratt, S. J. Blundell, T. Prokscha, A. Suter, J. Waizner, M. Garst, D. Grundler, G. van der Laan, C. Pfleiderer, T. Hesjedal

**Affiliations:** 1 Department of Physics, Clarendon Laboratory, University of Oxford, Oxford, OX1 3PU UK; 2 Lehrstuhl für Physik funktionaler Schichtsysteme, Technische Universität München, Physik Department, D-85748 Garching, Germany; 3 Centre for Materials Physics, Durham University, Durham, DH1 3LE UK; 4 Lehrstuhl für Topologie korrelierter Systeme, Technische Universität München, Physik Department, D-85748 Garching, Germany; 5 Magnetic Spectroscopy Group, Diamond Light Source, Didcot, OX11 0DE UK; 6 Laboratory for Muon Spin Spectroscopy, Paul Scherrer Institut, CH-5232 Villigen, Switzerland; 7 ISIS Facility, STFC Rutherford Appleton Laboratory, Chilton, Didcot, Oxfordshire OX11 0QX UK; 8 Institut für Theoretische Physik, Universität zu Köln, 50937 Köln, Germany; 9 Institut für Theoretische Physik, Technische Universität Dresden, 01062 Dresden, Germany; 10 Institute of Materials and Laboratory of Nanoscale Magnetic Materials and Magnonics, School of Engineering, École Polytechnique Fédérale de Lausanne, CH-1015 Lausanne, Switzerland

## Abstract

Chiral magnets are promising materials for the realisation of high-density and low-power spintronic memory devices. For these future applications, a key requirement is the synthesis of appropriate materials in the form of thin films ordering well above room temperature. Driven by the Dzyaloshinskii-Moriya interaction, the cubic compound FeGe exhibits helimagnetism with a relatively high transition temperature of 278 K in bulk crystals. We demonstrate that this temperature can be enhanced significantly in thin films. Using x-ray scattering and ferromagnetic resonance techniques, we provide unambiguous experimental evidence for long-wavelength helimagnetic order at room temperature and magnetic properties similar to the bulk material. We obtain *α*_intr_ = 0.0036 ± 0.0003 at 310 K for the intrinsic damping parameter. We probe the dynamics of the system by means of muon-spin rotation, indicating that the ground state is reached via a freezing out of slow dynamics. Our work paves the way towards the fabrication of thin films of chiral magnets that host certain spin whirls, so-called skyrmions, at room temperature and potentially offer integrability into modern electronics.

## Introduction

The exploration of new magnetic materials for nonvolatile memory is an important enterprise in the development of future information technology. High storage density and low power consumption are two of the most important criteria in the search for new systems. In this regard, materials hosting non-uniformly ordered magnetic phases are of great interest^1–4^ with the magnetic skyrmions being a prominent example^5–15^. Further candidates comprise spiral magnetic systems, in which the spins twist along one direction forming a long-wavelength, periodic modulation. Based on this state, several encoding schemes were proposed that could potentially be used in novel memory schemes, e.g., in the form of solitonic kinks^16, 17^, which intend to replace conventional ferromagnetic domain-based magnetic memory^18–21^.

The application potential of chiral spin order, however, is drastically hampered by the latter usually being a low-temperature phenomenon^22–25^, with Co-Mn-Zn alloys representing a recently discovered exception^26^. In the present study, we show that room-temperature helimagnetism overcoming these limitations is achieved in sputtered thin films of cubic FeGe by choosing appropriate substrates and growth conditions^27^.

FeGe belongs to the class of cubic chiral magnets crystallising in the noncentrosymmetric space group *P*2_1_3, which are also referred to as B20 compounds. Their magnetism is determined by the competition between exchange interactions and the Dzyaloshinskii-Moriya interaction. In combination with the weak magneto-crystalline anisotropies, a well-understood magnetic phase diagram evolves in bulk materials, comprising the helical, conical, and skyrmion lattice states^28^. Among this class of compounds, FeGe possesses the highest ordering temperature, *T*_*c*_ = 278 K^29, 30^, as compared to 29 K in MnSi^5, 31^, 170 K in MnGe^32, 33^, or 58 K in the insulating Cu_2_OSeO_3_^9, 34^.

The magnetic phase diagram is distinctly modified in thin films. Thinning bulk samples to thicknesses of the order of the helical wavelength (~70 nm in case of FeGe) drastically enlarges the phase pocket in which the skyrmion lattice exists^35, 36^. In thin-film/substrate systems, notably sputtered films of FeGe on Si(111), additional Hall contributions were observed across large parts of the phase diagram^37, 38^. Whether these contributions arise from topologically nontrivial objects, such as skyrmions, is still controversial.

In any case, the strain stemming from the lattice mismatch of substrate and film is a key ingredient when trying to address the magnetic properties of the film^39–42^. Note, however, that the ordering temperatures of all samples were similar to the bulk value.

The controversy partly originates from the fact that the unambiguous determination of the magnetic state in cubic chiral magnets can be a challenging task, in particular in thin film samples. Small-angle neutron scattering, a technique commonly used for bulk specimens^5, 30, 34, 43^, lacks the sensitivity necessary for the small sample volumes of thin films. Lorentz transmission electron microscopy, on the other hand, requires a careful thinning of the sample^9, 35, 44, 45^, interfering for instance with the strain inherent in thin film-substrate systems. Although polarised neutron reflectometry has been used to characterise the spin helix in MnSi thin films^46^, it is important to note that the technique only probes the structure of the helix motif and not the long-range ordered superlattice. In scanning probe microscopy, such as magnetic force microscopy^47, 48^ and scanning tunnelling microscopy^49^, the magnetic tip can influence the spin texture under investigation. Due to this lack of experimental techniques directly probing the magnetic structure, methods such as magnetometry, electrical transport, and Hall effect measurements are often used. While again being well-understood in bulk materials, in thin films these methods yield results that leave room for various interpretations^50–52^.

In our study of thin films of FeGe, we combine several experimental techniques mostly ignored so far. The key results are as follows. (i) The transition temperature of sputtered films can be systematically engineered and raised above room temperature, controlled by the growth conditions and the choice of substrate. (ii) By means of resonant elastic x-ray scattering (REXS), we determine an out-of-plane helimagnetic modulation, demonstrating the potential of this technique for the study of helimagnetic thin films (and bulk materials). (iii) Ferromagnetic resonance (FMR) measurements indicate that the collective excitations in our films are in excellent agreement with the well-understood behaviour of bulk material. (iv) Using transverse field muon-spin rotation (TF *μ*SR) we show that the ground state is reached by a freezing out of slow dynamics.

## Results and Discussion

### Structural and magnetic properties

B20-phase FeGe thin films were grown by magnetron sputtering, with the film thickness ranging between 40–200 nm.

As a substrate we use inert (001)-oriented MgO. This choice contrasts all previous work on thin films of MnSi^27, 46, 53^, FeGe^37, 38, 54^, and Fe_1−*x*_Co_*x*_Si^55^ grown by sputtering or molecular beam epitaxy, as these samples were always prepared on (reactive) Si(111) substrates. MgO, however, offers two major advantages: (i) the films can be deposited directly onto the substrate without the need for a seed layer, largely eliminating complications arising from the formation of a magnetically active interfacial layer^27^; (ii) while the use of Si substrates causes tensile strain in the FeGe films, the lattice mismatch between MgO (*a* = 4.13 Å) and FeGe (*a* = 4.70 Å) should lead to compressive strain and an enhancement of *T*_c_. For our measurements, we chose samples from two growth series that differ in the substrate temperature during preparation. The samples are characterised either by a low *T*_c_ of ~280 K, labelled FeGe^*L*^, or a high *T*_c_ of ~310 K, labelled FeGe^*H*^, respectively. Note that these transition temperatures are dependent only on the growth conditions and are largely independent of the film thickness in the range 40–200 nm. A plot of *T*_c_ as a function of growth temperature is shown in Supplementary Figure S3. The FeGe^*L*^ and FeGe^*H*^ samples were grown at a substrate temperature of 500 °C and 400 °C, respectively.

**Figure 1:**
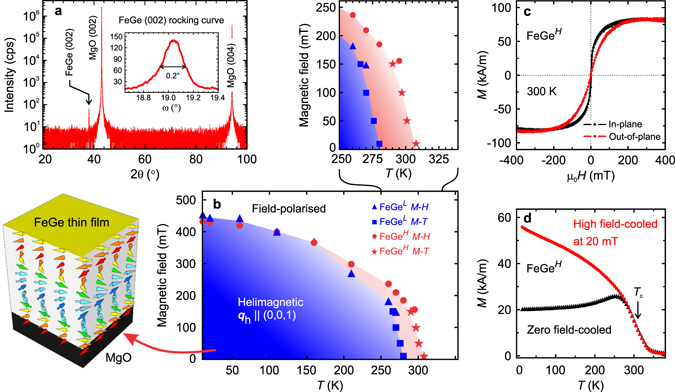
Structural and magnetic study of ~200-nm-thick FeGe films. (**a**) Out-of-plane x-ray diffraction showing a preferred (002) orientation of the FeGe film. The inset shows the rocking curve of the FeGe (002) peak. (**b**) Magnetic phase diagrams for FeGe^*L*^ and FeGe^*H*^ films. The blue and red shaded areas represent the phase space of the helimagnetic phase for the FeGe^*L*^ and FeGe^*H*^ films, respectively. A close-up of the phase space around the transition temperature is shown above. (**c**) Room-temperature magnetisation measurement with the field applied both in-plane and out-of-plane. (**d**) Temperature dependence of the magnetisation. The zero field-cooled curve is obtained by first cooling the sample in zero field from 300 K down to 10 K before measuring in an out-of-plane field of 20 mT while heating. The high field-cooled data are obtained by first cooling in an applied field of 500 mT down to 10 K, before measuring in an out-of-plane field of 20 mT while heating.

Figure 1a shows typical out-of-plane x-ray diffraction results for the as-grown FeGe thin films (FeGe^*L*^ shown here). Only the B20 FeGe (002) peak can be identified, which implies that the film is well-aligned with the substrate orientation. No additional Fe-Ge phases are found. The full width at half maximum of the rocking curve about the FeGe (002) peak is less than 0.2°, as shown in the inset of Fig. 1a, indicating a high crystalline quality.

As shown in Fig. 1d, the magnetisation–temperature profile for a 200 nm-thick FeGe^*H*^ sample resembles that found for FeGe bulk crystals, with a kink-like feature observed near the transition temperature in the zero-field-cooled curve implying a critical temperature *T*_c_ ≈ 310 K. (The divergence of field-cooled and zero field-cooled curves will be addressed below). The *M*-*H* loop measured at 300 K, shown in Fig. 1c, is indicative of an anisotropic magnetically ordered state (where the anisotropy arises due to the weakly locked cubic anisotropy of bulk crystals being altered in the thin film limit, giving rise to an easy-plane anisotropy). The saturation magnetisation at 20 K is 0.77 *μ*_B_/Fe, somewhat lower than the value reported for FeGe films by Porter *et al.* at *T* = 5 K^38^ and less than the bulk value of 1.0 *μ*_B_/Fe^56^. The combined phase diagrams for ~200 nm FeGe^*L*^ and FeGe^*H*^ films are shown in Fig. 1b. Inside the boundaries, the films are helimagnetically ordered with the spin helix propagating along the film normal (as shown on the left). The difference between the helimagnetic phase diagram for the FeGe^*L*^ (red area) and FeGe^*H*^ (blue area) films is highlighted in the close-up above. The magnetic phase boundaries were determined from *M*-*H* curves and their derivatives at different temperatures, as shown in Supplementary Figure S2a,b. The critical fields and magnetic phase boundaries are similar those previously reported for other B20 FeGe films^35, 37, 57^.

### Helical structure characterised by resonant elastic x-ray scattering

The magnetic ground state of the bulk cubic chiral magnets is a proper-screw-type spin spiral. It can be described by a one-dimensional harmonic model in which the spin rotates within a common plane that is perpendicular to the propagation direction, forming a periodic lattice structure that gives rise to magnetic Bragg reflections. However, the propagation direction wave vector **q**_*h*_ in thin film samples is found to be different from material to material^17, 40, 46, 51, 58^. For example, for MnSi thin films, polarised neutron reflectometry and theoretical studies suggest that the spin helix propagates along the film normal, due to the enhanced easy-plane magnetic anisotropy^40, 46^, while Lorentz transmission electron microscopy studies suggest that the helix is locked in-plane^51^. We performed REXS measurements on 200-nm-thick FeGe^*L*^ and FeGe^*H*^ films in order to determine the helix propagation direction and the periodicity of the helical lattice. REXS is a element-specific technique that can effectively probe long-range ordered, modulated magnetic structures. Within the soft x-ray region, the long-wavelength spin modulation wave vector can be unambiguously determined^59–61^.

**Figure 2:**
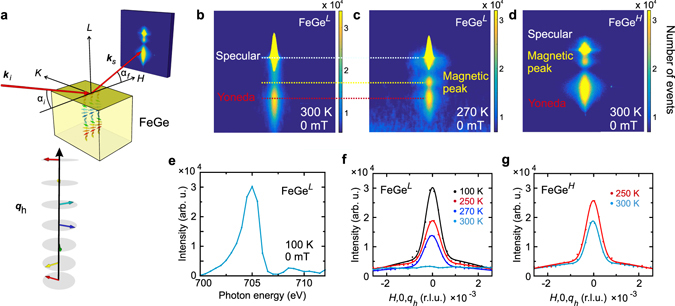
Resonant elastic x-ray scattering on FeGe films. (**a**) Illustration of the scattering geometry. The incident (scattered) x-ray wave vectors are labelled as **k**_*i*_ (**k**_*s*_), and the corresponding incident (outgoing) angle as *α*_*i*_ (*α*_*f*_). The photon energy is tuned to 705 eV near the Fe *L*_3_ edge. If **q**_*h*_ is along the FeGe film normal, a magnetic peak at (0, 0, *q*_*h*_) is expected, however, this requires small incident angles. (**b**,**c**) CCD images showing the magnetic contrast at 300 K and 270 K, at zero applied magnetic field for a FeGe^*L*^ film, with *α*_*i*_ = 2.1°. The specular reflectivity, Yoneda, and magnetics peaks are labelled. Note that the magnetic peak does not fulfil the perfect diffraction condition at this incident angle, therefore it is detached from the specular peak. Further data processing reveals that this peak corresponds to *q*_*h*_ = *L* = 0.0063 r.l.u. (**d**) CCD image showing the magnetic (0, 0, *q*_*h*_) peak for the FeGe^*H*^ sample at 300 K (*α*_*i*_ = 1.5°). Note that the magnetic peak is at a different position compared to (**c**) as *α*_*i*_ is different. (**e**) Photon energy-dependence of the magnetic peak at (0, 0, *q*_*h*_) across the Fe *L*_2,3_ resonance showing the magnetic origin of this peak. (**f**,**g**) Resonant *H*-scan about the magnetic satellite at different temperatures for a FeGe^*L*^ and FeGe^*H*^ sample, respectively.

Figure 2a shows the measurement geometry. The incident light, with wave vector **k**_*i*_, is tuned to the Fe *L*_3_ edge at 705 eV with *σ*-polarisation. The diffracted beam, with wave vector **k**_*s*_ (and a scattering wave vector **q** = **k**_*s*_ − **k**_*i*_), is captured either by a photodiode point detector or a CCD detector. If the modulated spin structure is along the [001] direction (as shown in Fig. 2a), it will give rise to a magnetic diffraction peak along the *L* direction at **q**_*h*_ in reciprocal space. As **q**_*h*_ is small, the grazing incident geometry is employed, with *α*_*i*_ scanning from 0° to 3.5°. However, various non-magnetic scattering processes will also occur in this geometry, such as (i) specular reflectivity that satisfies *α*_*i*_ = *α*_*f*_; (ii) the interference of reflections from the interface of the film (Kiessig fringes); and (iii) the Yoneda peak^62–64^ which is due to diffuse scattering and which satisfies *α*_*f*_ = *θ*_c_, where *θ*_c_ is the critical angle of total external x-ray reflection. Therefore, special care needs to be taken in order to separate the magnetic peak. This involves selecting a film thickness that is not a multiple of the helix pitch and also a systematic variation of parameters such as photon energy, sample temperature, and applied field. Figure 2b,c shows the CCD image for a FeGe^*L*^ sample at different temperatures, and Fig. 2d for a FeGe^*H*^ sample at 300 K. For the FeGe^*L*^ sample at 300 K no magnetic order is observed and only the specular reflection and the Yoneda peak can be seen on the CCD detector. When cooled just below *T*_c_, the magnetic diffraction peak appears corresponding to the helical wave vector **q**_*h*_. Note that for a perfect diffraction condition, the magnetic peak (0, 0, *q*_*h*_) always overlaps with the specular reflectivity, and is therefore indistinguishable on the detector image. However, in Fig. 2b,c, we intentionally offset the diffraction condition for the magnetic peak, leading to the separation between (0, 0, *q*_*h*_) and the specular. This peak corresponds to *L* = *q*_*h*_ = 0.0063 reciprocal lattice units (r.l.u.).

Reciprocal space scans along *H* at different temperatures for the FeGe^*L*^ sample are shown in Fig. 2f. Note that the non-zero background is due to either from specular or Yoneda peaks. We note first that only the modulation wave vector along *L* is observed, suggesting that the spins uniformly propagate along the film normal direction. This is in agreement with the conclusion reported in refs 17, 40, 46, in which the easy-plane anisotropy locks the propagation vector perpendicular to the film, regardless of its exact crystalline orientation. Secondly, we note that the corresponding real-space helical pitch is ~74.6 nm, in agreement with the bulk behaviour^35^. Finally, the photon energy scan on **q**_*h*_ across the Fe *L*_2,3_ edges, shown in Fig. 2e, confirms that the diffraction peak has an ordered magnetic origin. Note that if the resonance is charge related, a similar spectrum could be observed, however, in the case of FeGe the diffraction wave vector does not correspond to any structural peak. Therefore, we can exclude that this peak is due to charge enhancement.

Most significantly, the same magnetic peak that corresponds to the FeGe helical ground state is observed in the FeGe^*H*^ sample at room temperature, as shown in the CCD image in Fig. 2d and the *H*-scan in Fig. 2g (in contrast to the data for the FeGe^*L*^ sample shown in Fig. 2f), suggesting the successful realisation of room temperature helimagnetism in B20 compound thin films. We now turn to the dynamic behaviour of this system at different timescales, and the influence of domains.

### Dynamical properties measured by broadband resonance spectroscopy

**Figure 3:**
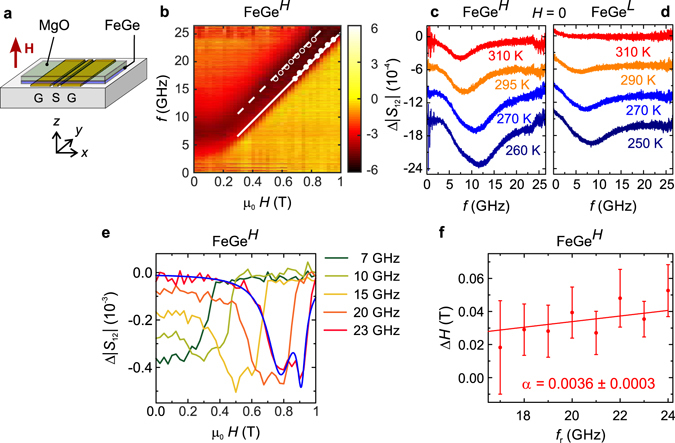
Broadband spectroscopy data. (**a**) The FeGe thin film sample is placed top-down onto the CPW consisting of a signal (S) and two ground (G) lines. The static field **H** is applied perpendicular to the thin film and the rf current applied to the conduction lines produces an rf field *h*_*x*_ along *x*. (**b**) Colour-coded microwave absorption Δ|*S*_12_| spectra at different magnetic fields recorded at 310 K on a FeGe^*H*^ sample. Darker regions correspond to stronger absorption. The dashed and solid lines correspond to a fit to PSSW modes, see text. Dots show fitted resonances (the error margin corresponds to the symbol size). (**c**,**d**) Zero-field absorption spectra at different temperatures recorded on a FeGe^*H*^ and FeGe^*L*^ sample, respectively. For the latter, no resonance is resolved above 290 K. Spectra are offset for clarity. The increased noise for *f* < 2.5 GHz and *f* > 22 GHz is attributed to the transmission properties of the microwave antenna. (**e**) Spectra at constant frequency, as a function of applied field at 310 K and exemplary fit of the sum of two Lorentzians (blue) to the 23 GHz data. (**f**) Field linewidth of the high-field mode (solid line in **b**) with linear fit for determining the intrinsic damping parameter *α*_intr_.

Broadband absorption spectroscopy was used to study spin excitations in FeGe thin films at microwave frequencies, as it has been successfully used for the investigation of the dynamics in bulk materials^65–68^. As shown in Fig. 3a, the film is placed directly on top of a coplanar waveguide (CPW). A static magnetic field *H* is applied perpendicular to the film. Using a vector network analyser, a radio-frequency alternating magnetic field is induced by the CPW and the absorption by spin-precessional motion in the sample is recorded.

Figure 3b shows a colour-map of the microwave absorption of a FeGe^*H*^ sample as derived from frequency sweeps in different applied magnetic fields 0 ≤ *μ*_0_ *H* ≤ 1 T at 310 K. In the region below 0.1 T, we resolve a resonance whose frequency decreases as the external field *H* increases. This is the expected behaviour for a non-collinear ordered spin state with *H* below the critical field. At higher applied magnetic fields, the resonance frequency increases linearly with *H*, and the absorption becomes more intense as the magnet become polarised. The dashed and solid lines are obtained by a fit to perpendicular standing spin wave (PSSW) modes of the film (see Supplementary section S4 for details). Figure 3c,d shows the temperature evolution of spectra for the FeGe^*H*^ and FeGe^*L*^ samples, respectively, taken at *H* = 0. In both cases, the signal intensity diminishes with increasing temperature and vanishes for sample FeGe^*L*^ above 290 K, as it reaches the paramagnetic state. For the sample FeGe^*H*^, the signal persists to higher temperatures indicating that magnetic order is still present above *T* = 300 K on the timescale of these measurements.

For the FeGe^*H*^ sample we evaluated the intrinsic damping parameter *α*_intr_ from the spectra shown in Fig. 3e. These show the magnetic resonance curves taken at different fixed frequencies *f*′ as a function of the applied magnetic field *H*. The field *H* was varied in finite steps. We used the sum of two Lorentzians (blue line) to fit the resonant curves that feature double-peak structures. The resonance fields *H*_r_ (dots) extracted for the different frequencies *f*′ are summarized in Fig. 3b and agree very well with both the modelling (lines) and the resonance features resolved in the frequency-dependent spectra taken at fixed *H* (dark colour in the colour-coded data depicted in the background).

To extract *α*_intr_ we consider the full-width-at-half-maximum that is given by the fitted Lorentzian peak that describes the main resonance with high *H*_r_. Since we detect the magnitude of the scattering parameter using the vector network analyser, we divide each full-width-at-half-maximum value by $\sqrt{3}$ to obtain the linewidth Δ*H* that is conventionally considered and extracted from the imaginary part of absorption spectra^69^. We summarize the linewidth values Δ*H* at 310 K as a function of the resonance frequency *f*_r_ in Fig. 3f. We observed Δ*H* of about 20 mT at 17 GHz. Considering ref. 70 we fitted a linear function *μ*_0_Δ*H* = *μ*_0_Δ*H*_0_ + 4*πα*_intr_ *f*_r_/*γ* to the given data in Fig. 3f, where *γ* is the gyromagnetic ratio. This function (not shown) provided a negative Δ*H*_0_ = (−28 ± 27) mT containing a large error. A negative inhomogeneous broadening was not reasonable. We thus extracted the damping parameter *α*_intr_ = 0.0036 ± 0.0003 from the slope of a linear fit (red line) by assuming Δ*H*_0_ = 0, which represented the smallest reasonable inhomogeneous broadening and provided the upper limit of *α*_intr_.

Beg *et al.* ^71^ reported a damping parameter *α*_intr_ = 0.28 ± 0.02 obtained on a Si/FeGe film capped with Ge. Turgut *et al.* ^72^ observed a linewidth Δ*H* of about 58 mT at 7 GHz and obtained *α*_intr_ = 0.021 ± 0.005 as a minimum value at 263 K. They investigated FeGe films grown on undoped Si[111] wafers by magnetron sputtering. Our value *α*_intr_ = 0.0036 ± 0.0003 extracted from a MgO(100)-grown FeGe film is smaller by a factor of about 6. This value is also about ten times smaller than the one reported for bulk metallic MnSi at 28 K by Schwarze *et al.* ^68^. To our knowledge, the film investigated here shows the smallest *α*_intr_ reported for FeGe so far. We note that this damping parameter is on the order of metallic permalloy (Ni_80_ Fe_20_) that is exploited in spin-based electronics.

### Slow dynamical properties studied with muon-spin rotation

Low-energy TF *μ* ^+^SR is a sensitive probe of the local magnetic field distribution in a thin magnetic film and of its dynamics. In a TF *μ* ^+^SR experiment, spin polarised muons are implanted in the bulk of a material in the presence of a magnetic field *B*_0_ = *μ*_0_ *H*_0_, which is applied perpendicular to the initial muon spin direction. The low energy muons stop in the thin film of FeGe where they precess about the total local magnetic field *B* = *μ*_0_ *H* at the muon site, with frequency *ω* = *γ*_*μ*_ *B*, where *γ*_*μ*_ = 2*π* × 135.5 MHz T^−1^. The observed property of the experiment is the time evolution of the muon spin polarisation *P*_*x*_(*t*), which allows the determination of the distribution *p*(*B*) of local magnetic fields across the sample volume via ${P}_{x}(t)={\int }_{0}^{\infty }{\rm{d}}Bp(B)\cos ({\gamma }_{\mu }Bt+\varphi )$ where the phase *ϕ* results from the detector geometry.

**Figure 4:**
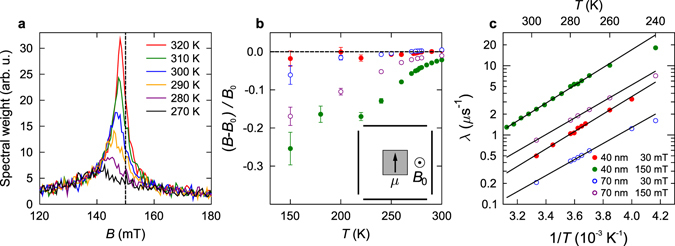
Transverse muon-spin rotation data. (**a**) Fourier spectra measured in an applied field of *B*_0_ = 150 mT for a 40-nm-thick film of FeGe^*L*^. Evolution with temperature of (**b**) the diamagnetic shift and (**c**) the relaxation rate *λ* for the two FeGe films at two values of the applied field *B*_0_. Temperature-activated behaviour is evident above *T* = 260 K, as described in the text.

TF *μ* ^+^SR measurements were carried out on FeGe^*L*^ films to study the slow dynamical properties. Figure 4a shows fairly symmetric Fourier spectra obtained from the 40-nm-thick film, measured on cooling in a fixed, applied magnetic field *B*_0_ = 150 mT. A diamagnetic shift is evident, with the peak field decreasing with decreasing temperature. Similar behaviour is seen in analogous measurements of MnSi films^73^, caused by a Knight shift, which is likely also to be the case here. The peak is seen to broaden considerably with decreasing temperature, becoming effectively wiped out on cooling though the region 200 ≤ *T* ≤ 260 K. The spectra in each detector were fitted in the time-domain to a polarisation function ${P}_{x}(t)=\exp (-\lambda t)\cos ({\gamma }_{\mu }Bt+{\varphi }_{i})$, where *ϕ*_*i*_ is a phase shift reflecting the position of the particular detector being considered. The relaxation rate *λ* and the diamagnetic shift Δ*B* = (*B* − *B*_0_)/*B*_0_ is shown for the 40 nm film and applied field *B*_0_ in Fig. 4b.

It is notable that we do not observe any discontinuity in the behaviour of *λ* nor Δ*B* as the temperature is varied through the nominal ordering temperature at *T*_c_ ≈ 280 K. This is unlike the case of MnSi^73^, where the analogous quantities showed sharp changes at the bulk and film transitions. In contrast, for FeGe the fitted parameters appear to vary quite smoothly from 320 K down to ~200 K. This means that instead of a sharply defined ordering temperature, a significant broadening is observed, which points to the development of a distribution of large, quasistatic local magnetic fields as the temperature is lowered below 260 K.

The evolution of the relaxation rate as a function of temperature, *λ*(*T* > 260 K), at fixed field is well-described by an activated behaviour in the form of

1 $$\lambda (T)={\lambda }_{0}\exp ({\rm{\Delta }}E/{k}_{{\rm{B}}}T),$$

where Δ*E* is the characteristic activation energy. The extracted values of Δ*E* are given in Table 1 for each film. The activation energy at *B*_0_ = 150 mT is found to be Δ*E* ≈ 0.26 eV for the 40 nm film and Δ*E* ≈ 0.23 eV for the 70 nm film. One possible cause of temperature-activated relaxation is muon hopping between equivalent crystallographic sites. By comparison with MnSi, which has the same *P*2_1_3 space group as FeGe, electronic structure calculations^74^ (Bonfà, P. private communications (2016)) suggest that the energetic barrier to hopping is greater than 1.1 eV, i.e., far larger than the measured activation barriers in FeGe of ~0.2 eV. Further, zero-field *μ* ^+^SR measurements on MnSi^75^ demonstrate that there is no dynamics due to hopping at *T* = 285 K, where a static Kubo-Toyabe function is observed. Even given the slightly larger lattice constants in FeGe (4.70 Å) compared to MnSi (4.56 Å) it is hard to believe that the barrier to muon hopping would vary substantially.

**Table 1:** Table of parameters from fits of the relaxation rate *λ* to the activated behaviour in Eq. (1).

Sample	Film thickness	*B*_0_	*λ*_0_	Δ*E*
	(nm)	(mT)	(10^−5^ *μ*s^−1^)	(eV)
FeGe^*L*^	40	150	12(1)	0.26(1)
		50	1.4(2)	0.29(1)
		30	3.3(4)	0.25(1)
FeGe^*L*^	70	150	8.8(2)	0.24(1)
		30	3.0(4)	0.23(1)

Instead, the activated temperature dependence is more likely to be caused directly by the magnetic state of FeGe. In general, relaxation of the *μ* ^+^SR signal is caused by static magnetic disorder or dynamics in the local field distributions. The fact that the relaxation is exponential, with a rate that falls as the temperature increases suggests that we are in the fast fluctuation limit of dynamic fluctuations and that the correlation time *τ* is dropping with increasing temperature. If this is the case then, using the Abragam function^76^, we expect the relaxation rate to be given by *λ* = *γ*_*μ*_^2^〈(*B* − 〈*B*〉)^2^〉*τ*. For the 40 nm film, at an applied field of *B*_0_ = 150 mT and a temperature *T* ≈ 280 K, we estimate from Fig. 4b that (*B* − *B*_0_)/*B*_0_ = −0.05 (which we take as measure of the scale of *B* − 〈*B*〉) and [from Fig. 4c] *λ* ≈ 5 *μ*s^−1^. We can then make a crude estimate of a typical correlation time in this temperature regime of *τ* ≈ 0.1 *μ*s (corresponding to an attempt frequency of the activated dynamics of *ν*_0_ = *τ*(*T*)^−1^ exp(Δ*E*/*k*_B_ *T*) ≈ 5 × 10^11^ s^−1^). The correlation time would be expected to increase to *τ* ≈ 0.2 *μ*s as the temperature is lowered towards 260 K.

We therefore infer that the magnetism in FeGe films involves dynamics in the underlying magnetic field distribution on the muon (microsecond) timescale. These dynamics are present well above the nominal ordering temperature of these film samples (*T*_c_ ≈ 280 K) and gradually freeze out as the temperature is lowered until a more static state is achieved below 260 K. It is likely that such a freezing out (as opposed to a sharply defined ordering transition) occurs on several timescales, and we observe that part of the dynamics with spectral weight in the muon time window that is set by *γ*_*μ*_. We note that this effect is not seen by the x-ray nor the FMR measurements which, having a measurement timescale much faster than the muon (ns vs. *μ*s timescale), effectively take an average of snap-shots of an ordered magnetic system. The dynamically varying fluctuations seen by the muon probe are therefore most probably related to collective fluctuations of large regions of ordered spins such as domains. The activation energy Δ*E* would then correspond to the energy barrier to domain wall motion. We note also that Δ*E* is larger in the thinner film sample, consistent with domain motion requiring more energy in a more confined geometry. It has recently been suggested that FeGe hosts spontaneous collective spin movements^77^ in the form of thermally-driven collective jumps in the spin system. A similar scenario would explain our *μ* ^+^SR measurements as it provides a mechanism for the thermally activated dynamics that we observe. On the other hand, as our films are thinner than a helical wavelength, and as they are not monochiral, magnetic frustration could occur at the grain boundaries, leading to glassy magnetic behaviour as was observed in MnSi by Karhu *et al.* ^78^. It is also possible that the dynamics we observe are consistent with the jamming behaviour of domains previously observed in a spiral antiferromagnet^79^. Although diffraction techniques are sensitive to the long-range helically ordered regions of FeGe, the muon, being a local probe, will decorate the ordered regions and the grain boundaries or defects and is therefore sensitive to changes in the field distribution around these boundaries.

Our picture of the dynamics is consistent with the *M*-*T* data (Fig. 1d), where we measure a splitting of the field-cooled and zero field-cooled curves around 260 K, where the muon signal begins to be wiped out owing to freezing. This splitting is typical of a freezing of the magnetic moments at low temperature, and reflects the fact that, after cooling in zero field to a region where the spins are statically frozen, we obtain a configuration which is less susceptible to magnetisation than occurs if the frozen state is achieved in an applied field^80^. It is notable that the splitting is found in magnetometry on a FeGe^*H*^ sample (while the muon measurements were made on a FeGe^*L*^ sample), suggesting that the freezing behaviour is intrinsic to FeGe.

## Summary and Conclusions

Turning to the question of the presence of skyrmions in FeGe thin films, we note that the wipe-out of the *μ*SR signal occurs below the temperatures where the skyrmion density was found to be large^44^, and in MnSi below the temperature at which the topological anomaly was found^51, 73^. It may be that skyrmions are the defects that cause collective jumps in the domain wall pattern in FeGe, recently observed by magnetic force microscopy^77^. Further, we note that we can exclude the existence of the hexagonally ordered skyrmion lattice phase that is characteristic in bulk FeGe, which can be clearly distinguished by REXS from any other magnetic phase^60, 61^. However, single skyrmions, or a disordered skyrmion phase coexisting along with the helical phase, would not lead to a diffraction pattern in REXS. Such a scenario would be consistent with the broadening of the FMR peak. Also, it has to be stressed that in our study, using MgO as a substrate, FeGe is presumably in a different strain state compared to previous work on FeGe films on Si(111)^37, 38, 54^, which is known from other B20 systems to lead to a different magnetic behaviour^46^.

In summary, we have demonstrated the growth of the B20 compound FeGe on a non-Si(111) substrate, thereby realising robust room temperature helimagnetism, as confirmed by magnetometry, REXS, and FMR. Our *μ* ^+^SR measurements reveal that the ordered state in the Fe hosts dynamic fluctuations below the ordering temperature, consistent with slowly fluctuating domains and that the ground state is achieved via a freezing out of these slow dynamics on cooling.

Sputtered films are ideal candidates for chiral spin device applications due to their ease of fabrication and compatibility with existing processes for magnetic devices. At the same time a small spin-wave damping parameter *α*_intr_ is obtained. The choice of substrate, and engineering of the interface^27^ and surface^81^, appears to be crucial for enabling the exploitation of complex magnetic textures, such as helimagnets and skyrmions. As a next step, nanopatterned FeGe films will be investigated, which promise the emergence of the skyrmion lattice phase owing to their constrained geometry^82^.

## Methods

### Sample preparation

The thin film samples were prepared by magnetron sputtering from a stoichiometric FeGe target in a home-built, two-chamber UHV deposition system. The base pressure was 5 × 10^−9^ mbar. For the DC sputtering process we used Ar as the sputtering gas. Prior to growth, the 10 × 10 mm^2^ and 1″-diameter MgO(001) substrates were degreased in boiling solvents and subsequently annealed in ultra-high vacuum for up to 8 h to get a well-defined surface. For the growth study, the substrate temperature was varied between room-temperature and 700 °C and the growth rate between 0.5 and 2 Å/min, as monitored by an *in-situ* quartz-crystal microbalance and *ex-situ* x-ray reflectivity (XRR), as shown in Supplementary Figure S1. After growth, the samples were annealed for up to 8 h at 400 °C.

### Structural characterisation

X-ray diffraction (XRD) and XRR measurements were performed on a Bruker D8 Discover x-ray diffractometer (Cu *Kα*_1_ radiation). The incident optics were set with a Ge (220) 2-bounce monochromator, 2.5° Soller slits and 1 mm beam mask. The receiving optics used 2.5° Soller slits arriving at either an area detector (XRD) or a scintillator counter (rocking curves). XRR was used to determine the thicknesses as indicated in the paper, as well as the surface roughness, which was found to be between 0.9 and 2 nm.

### Magnetometry

Magnetic measurements were carried out on a Quantum Design^TM^ superconducting quantum interference device (SQUID) vibrating sample magnetometer (VSM). Measurements were performed with the magnetic field applied in-plane and out-of-plane, in a temperature range between 10 and 300 K, as shown in Supplementary Figure S2.

### Resonant elastic x-ray scattering (REXS)

The resonant soft x-ray scattering experiments were carried out in the UHV diffractometer RASOR^83^ on beamline I10 at the Diamond Light Source (UK). The as-grown thin film samples were mounted on a cold-finger cryostat and their azimuthal angle was precisely aligned for subsequent resonant x-ray scattering measurements. The incident soft x-ray beam was tuned to near the *L*_3_ edge of Fe of 705 eV. The experimental setup is illustrated in Fig. 2a and more details can be found in refs 60, 61.

### Broadband microwave resonance spectroscopy

The measurements used a coplanar waveguide (CPW) with a width of the inner conductor of *w*_*s*_ = 20 *μ*m and was contacted with two microwave probes in a variable temperature probe station. A superconducting magnet supplied the static external field **H**. To ensure a well-defined magnetic history of the sample, we recorded frequency spectra starting from high fields. The data were recorded with an Agilent PNA N5222A vector network analyser (VNA), operating in the range from 10 MHz to 26.5 GHz. We applied a difference technique to enhance the signal to noise ratio in that we subtracted a reference spectrum $|{S}_{12}^{{\rm{ref}}}(f)|$, recorded at 1.3 T, from all subsequent spectra. For more details on the experimental setup and the measurement technique, see ref. 68 and Supplementary Information therein.

### *μ*SR spectroscopy

Transverse field (TF) muon-spin rotation (*μ* ^+^SR) measurements were made on FeGe thin film samples using the Low Energy Muon (LEM) beamline at S*μ*S in Villigen, Switzerland^84^. The LEM beamline produces very slow, spin-polarised, positive muons with a mean energy of about 15 eV, which can be used as a source of a tertiary beam with tuneable energy between 0 and 30 keV, suitable to be stopped in a thin sample^85, 86^. For the measurement the sample was glued to an Ag backing plate with applied magnetic fields directed perpendicular to the surface of the sample. Measurements were made on two FeGe films (40 nm and 70 nm thick).

## Electronic supplementary material

Supplementary Materials
