# Supplementary material for: Room-temperature helimagnetism in FeGe thin films
Source: Sci Rep. 2017 Mar 9;7:123. doi: 10.1038/s41598-017-00201-z (PMC5427977; doi:10.1038/s41598-017-00201-z)
Supplement: Supplementary file 1 — Supplementary Materials [file 41598_2017_201_MOESM1_ESM.pdf]

Room-Temperature Helimagnetism in FeGe Thin Films Supplementary Information

S. L. Zhang,[1] I. Stasinopoulos,[2] T. Lancaster,[3] F. Xiao,[3] A. Bauer,[4] F. Rucker,[4] A. A. Baker,[1,5] A. I. Figueroa,[5] Z. Salman,[6] F. L. Pratt,[7] S. J. Blundell,[1] T. Prokscha,[6] A. Suter,[6] J. Waizner,[8] M. Garst,[8,9] D. Grundler,[2,10] G. van der Laan,[5] C. Pfleiderer,[4] and T. Hesjedal[1]

[1)] *Department of Physics, Clarendon Laboratory, University of Oxford, Oxford, OX1 3PU, United Kingdom*

[2)] *Lehrstuhl für Physik funktionaler Schichtsysteme, Technische Universität München, Physik Department, D-85748 Garching, Germany*

[3)] *Centre for Materials Physics, Durham University, Durham, DH1 3LE, United Kingdom*

[4)] *Lehrstuhl für Topologie korrelierter Systeme, Technische Universität München, Physik Department, D-85748 Garching, Germany*

[5)] *Magnetic Spectroscopy Group, Diamond Light Source, Didcot, OX11 0DE, United Kingdom*

[6)] *Laboratory for Muon Spin Spectroscopy, Paul Scherrer Institut, CH-5232 Villigen, Switzerland*

[7)] *ISIS Facility, STFC Rutherford Appleton Laboratory, Chilton, Didcot, Oxfordshire, OX11 0QX, United Kingdom*

[8)] *Institut für Theoretische Physik, Universität zu Köln, 50937 Köln, Germany*

[9)] *Institut für Theoretische Physik, Technische Universität Dresden, 01062 Dresden, Germany*

[10)] *Institute of Materials and Laboratory of Nanoscale Magnetic Materials and Magnonics, School of Engineering, École Polytechnique Fédérale de Lausanne, CH-1015 Lausanne, Switzerland*

S1. SAMPLE PREPARATION

The Fe-Ge binary alloy system has a complex equilibrium phase diagram, whereby a large variety of phases exist with different stoichiometries, structural, and magnetic properties. We performed thin growth by magnetron sputtering using a stoichiometric (Fe:Ge=1:1) target. Except for the desired B20 phase, the most probable impurity phases to expect for our growth conditions are the B35 and the monoclinic phase. Both are antiferromagnetic, providing good magnetic contrast with the B20 phase.

FeGe thin film samples were grown in a home-built, two-chamber UHV magnetron sputtering system with a base pressure of 5×10^{-9} mbar. The MgO substrates were degreased prior to loading as described in the main text, followed by a high-temperature anneal in UHV for up to 8 h. The FeGe films are sputtered with an Ar partial pressure of 6×10^{-3} mbar, using a DC power of 40 W. The substrate temperature was kept at 400°C for the growth of the FeGe^H samples. A plot of the dependence of the magnetic transition temperature as a function of growth temperature is shown in Fig. S4. The thickness is monitored by an *in-situ* quartz crystal microbalance, and *ex-situ* using x-ray reflectivity (XRR), as shown in Fig. S1. Subsequent to the growth, the samples are annealed in UHV at 400°C for 4 h.

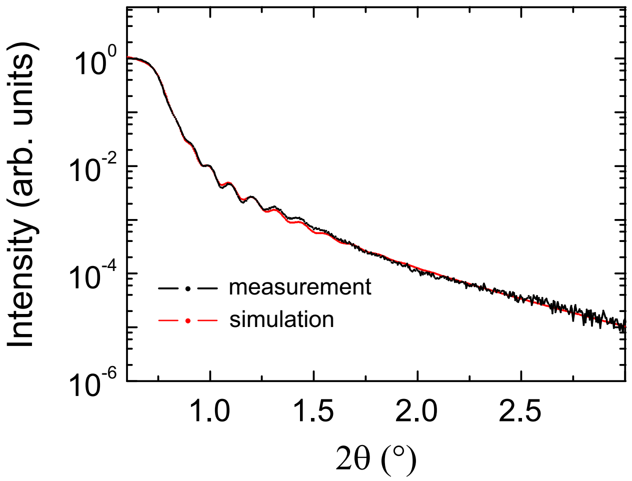

FIG. S1. X-ray reflectivity data for a typical FeGe film. The film was grown at 400°C on a 1"-diameter MgO(001) wafer. The numerous Kiessig fringes are indicative of well-defined interfaces. The data were fitted using the Parratt algorithm (red line). The thickness of this particular film is 65.2 nm (nominally 70 nm), with a root-mean-square roughness of 1-2 nm. This film was used for transverse field muon-spin rotation measurements.

S2. STRUCTURAL PROPERTIES

Based on the high-resolution out-of-plane x-ray diffraction (XRD) measurements (example shown in Fig. 1 in the main text), we are able to extract the FeGe(002) d-spacing of that film, resulting in a lattice constant of 4.6480 Å. Given that the bulk FeGe lattice constant is 4.70 Å, it can be concluded that the compressive strain is $\sim$1.1%.

Apart from the determination of the out-of-plane lattice parameters by XRD, we studied the perfection of the interfaces, and the thickness of the epitaxial films, using x-ray reflectivity. XRR scans were carried out on a D5000 diffractometer using Cu $K\alpha_1$ radiation. The reflectivity data was fitted using the Parratt32 algorithm[1]. An example of typical XRR data for these films is shown in Fig. S1.

In order to determine the crystalline relationship between film and substrate, we performed three-dimensional reciprocal space mapping using low-resolution XRD. The projected pole figure (see Fig. S2) shows the distribution of the MgO {113} (red) and FeGe {112} (black) peaks projected onto the hk-plane (see blue arrows), viewed along the l-direction. The four-fold symmetric MgO {113} substrate peaks are sharp, as expected. On the other hand, the FeGe {102} film peaks are diffuse. Their $|q|$-values remain constant and the overall pattern is roughly four-fold symmetric. Thus, it can be concluded that the FeGe film is textured and lacking in-plane orientation, with a perfect (002) out-of-plane orientation.

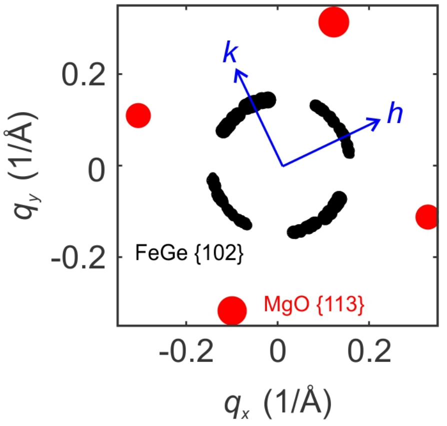

FIG. S2. Reciprocal space map of a typical FeGe film grown at 400°C on MgO(001) wafer. The MgO {311} and FeGe {210} peaks are labelled in red and black, respectively.

S3. MAGNETIC PROPERTIES

The magnetic properties of the films were determined using a superconducting quantum interference device (SQUID) magnetometer with a vibrating sample magnetometer module (Quantum Design). Magnetisation measurements were performed as a function of temperature, for the field applied in-plane and out-of-plane, in a temperature range between 10 and 300 K.

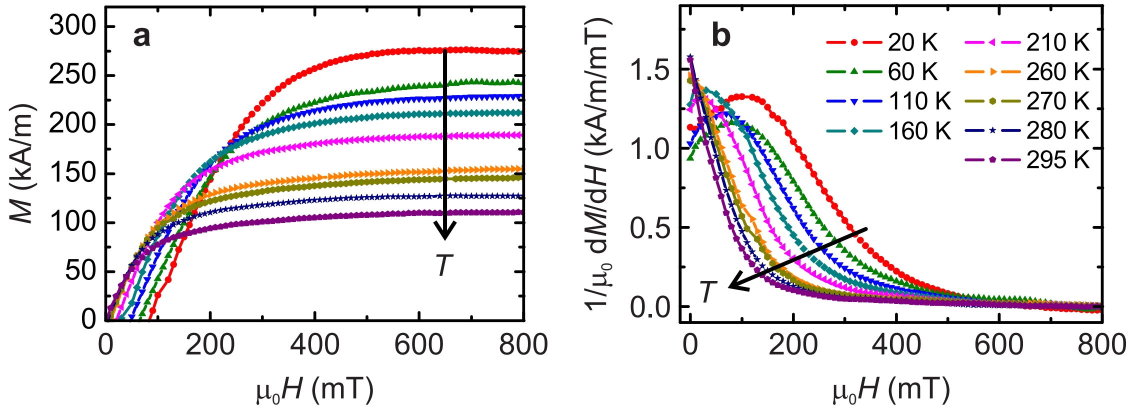

FIG. S3. Magnetic study of a ∼200 nm-thick FeGe film. (**a**) Magnetisation as a function of out-of-plane field at different temperatures. The legend is shown in (b). (**b**) Susceptibility as a function of field at various temperatures, derived from (a).

Figure S3a shows the $M(H)$ magnetisation curves taken in an out-of-plane field, after field-cooling from 300 K to the indicated temperature in an applied field of 2 T. The diamagnetic background stemming from the MgO substrate was subtracted by a linear fit to the high-field data. The shape of the curves is consistent with previous studies on FeGe. The susceptibility $1/\mu_0 \cdot dM/dH$, derived from the data shown in (a), is presented in Fig. S3b.

The magnetic transition temperature T_c, obtained from determining the minimum in the differentiated $M(T)$ plots, varies with the temperature at which the FeGe film was grown, as plotted in Fig. S4. Within reasonable limits, T_c is independent of the annealing time and temperature.

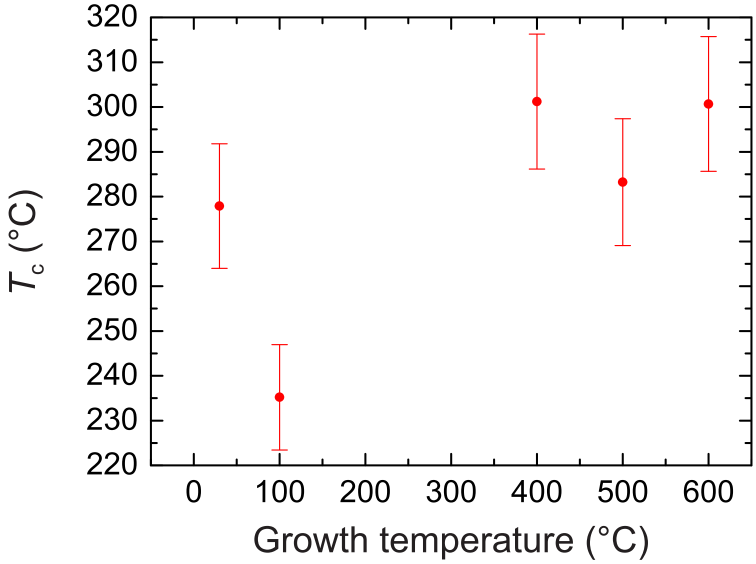

FIG. S4. Dependence of T_c of FeGe films as a function of growth temperature. The error bars indicate the variation of T_c for samples grown at the same temperature.

S4. FMR RESONANCES

For the interpretation of the FMR data we use the free energy functional for a chiral magnetic film $F = F_0 + F_{\text{dip}}$ with $F_0 = \int d\vec{r}\mathcal{F}_0$ and

$$\mathcal{F}_0 = A\Big((\nabla_i \hat{n}_j)^2 + 2Q\hat{n}(\nabla \times \hat{n})\Big) + K\hat{n}_z^2 - \mu_0 M_s \hat{n}\vec{H} \quad , \tag{1}$$

where the unit vector $\hat{n}(\vec{r}, t)$ represents the orientation of the local magnetisation, A is the exchange stiffness constant, K is the magnetic anisotropy, M_s is the saturation magnetisation, and $\vec{H}$ is the applied magnetic field. The second term is the Dzyaloshinskii-Moriya interaction parametrised by the helix pitch vector Q. The remaining term F_{dip} describes the dipolar interaction of the magnetisation field at finite momenta $\vec{k}$,

$$F_{\text{dip}} = \frac{\mu_0 M_s^2}{2V} \sum_{\vec{k}} \frac{(\hat{n}(\vec{k})\vec{k})(\vec{k}\hat{n}(-\vec{k}))}{k^2} \tag{2}$$

with $\hat{n}(\vec{k}) = \int d\vec{r}\hat{n}(\vec{r})e^{-i\vec{k}\vec{r}}$ and the volume V.

We consider the magnetic field applied perpendicular to the film along the z-axis, $\vec{H} = H\hat{z}$. The ground state is obtained by minimizing the energy functional. There is a phase transition between the conical phase and the field-polarised phase at the critical field $H_{c2,z} = \frac{\mathcal{D}Q^2}{g\mu_B\mu_0} + \frac{2K}{\mu_0 M_s} + M_s$ where $\mathcal{D} = 2Ag\mu_B/M_s$, see Ref. 2. The boundary conditions at the film

surfaces[3],

$$\partial_z \hat{n} - Q\hat{z} \times \hat{n} = 0 \tag{3}$$

must be fulfilled by the vector $\hat{n}$, from which follow the boundary conditions for the spin waves. The Dzyaloshinskii-Moriya interaction, Q, results in an effective pinning[4] of the magnetisation at the surface.The boundary condition might be modified in case of additional surface-pinning potentials which we do not consider here.

The magnon excitation spectrum is derived in the standard linear spin-wave approximation following Refs. 5 and 6. The spin-wave excitation is parametrised in terms of a complex wave function ψ,

$$\hat{n} = \hat{e}_3 \sqrt{1 - 2\frac{g\mu_B}{M_s}|\psi|^2} + \sqrt{\frac{g\mu_B}{M_s}} \Big(\psi \hat{e}_+ + \psi^* \hat{e}_-\Big) \tag{4}$$

where $\hat{e}_\pm = \frac{1}{\sqrt{2}}(\hat{e}_1 \pm i\hat{e}_2)$. The unit vectors $\hat{e}_i$ with $i = 1, 2, 3$ are orthonormal $\hat{e}_i\hat{e}_j = \delta_{ij}$ and the third component is identified with the equilibrium configuration $\hat{e}_3 \equiv \hat{n}_{\rm eq}$. Expanding the equation of motion $\partial_t \hat{n} = -\frac{g\mu_B}{\hbar}\hat{n} \times \vec{B}$ with $\vec{B} = -\frac{1}{M_s}\frac{\delta F}{\delta \hat{n}}$ in first order in the wave function ψ, one obtains an effective wave equation for the spin waves.

In the following, we discuss the result for the field-polarised phase, $H > H_{c2,z}$, where the magnetisation is field-polarised and $\hat{n}_{\rm eq} = \hat{z}$. We limit ourselves however to a discussion of magnon excitations homogeneous within the plane of the film, i.e., with vanishing in-plane wavevector, $\vec{k}_\perp = 0$. The stationary wave equation then reduces to (for $\vec{k}_\perp = 0$)

$$i\hbar\omega\psi_\omega(z) = \left[\mathcal{D}(-i\partial_z - Q)^2 + g\mu_B\mu_0(H - H_{c2,z})\right]\psi_\omega(z). \tag{5}$$

In addition, it follows from Eq. (3) that the wave function must obey the boundary condition

$$(-i\partial_z + Q)\psi_\omega(z)\Big|_{\rm surface} = 0. \tag{6}$$

For a film with surfaces located at $z = 0$ and $z = d$ with the film thickness d, the normalised eigenfunctions consistent with the boundary condition are given by

$$\psi_p(z) = \frac{1}{\sqrt{d/2}} \cos\Big(\frac{\pi p}{d} z\Big) e^{-iQz} \tag{7}$$

with the integer quantum number $p = 0, 1, 2, 3, ...$ They specify the so-called perpendicular standing spin wave (PSSW) modes[4] of a film with Dzyaloshinskii-Moriya interaction. The corresponding discrete energy spectrum is given by

$$\hbar\omega_p = \mathcal{D}\Big(\frac{\pi p}{d}\Big)^2 + g\mu_B\mu_0(H - H_{c2,z}). \tag{8}$$

Note that the PSSW modes differ from a conventional ferromagnet[7] due to the oscillating factor e^{-iQz} as was pointed out in Ref. 8.

In Fig. 3 of the main text, we have assumed that at large fields the two modes seen experimentally correspond to $p = 0$ and 1. With these assumptions, the resulting fit yields $\mu_0 H_{c2,z} \approx 0.05$ T and $\frac{\mathcal{D}\pi^2/d^2}{g\mu_B\mu_0 H_{c2,z}} \approx 3$ with $g = 1.9$. We have refrained from fitting the data at low fields to a theory for the conical phase. The small value for the critical field indicates that the film at zero field is already close to the phase transition between the conical and the paramagnetic phase hampering a quantitative comparison with theory.

REFERENCES

[1] L. G. Parratt, "Surface studies of solids by total reflection of x-rays," Phys. Rev. **95**, 359 (1954).

[2] E. A. Karhu, U. K. Rößler, A. N. Bogdanov, S. Kahwaji, B. J. Kirby, H. Fritzsche, M. D. Robertson, C. F. Majkrzak, and T. L. Monchesky, "Chiral modulations and reorientation effects in MnSi thin films," Phys. Rev. B **85**, 094429 (2012).

[3] S. Rohart and A. Thiaville, "Skyrmion confinement in ultrathin film nanostructures in the presence of Dzyaloshinskii-Moriya interaction," Phys. Rev. B **88**, 184422 (2013).

[4] S. O. Demokritov, B. Hillebrands, and A. N. Slavin, "Brillouin light scattering studies of confined spin waves: Linear and nonlinear confinement," Phys. Rep. **348**, 441–489 (2001).

[5] M. Kugler, G. Brandl, J. Waizner, M. Janoschek, R. Georgii, A. Bauer, K. Seemann, A. Rosch, C. Pfleiderer, P. Böni, and M. Garst, "Band structure of helimagnons in MnSi resolved by inelastic neutron scattering," Phys. Rev. Lett. **115**, 097203 (2015).

[6] T. Schwarze, J. Waizner, M. Garst, A. Bauer, I. Stasinopoulos, H. Berger, C. Pfleiderer, and D. Grundler, "Universal helimagnon and skyrmion excitations in metallic, semiconducting and insulating chiral magnets," Nat. Mater. **14**, 478 (2015).

[7] Y. Li and W. E. Bailey, "Wave-number-dependent Gilbert damping in metallic ferromagnets," Phys. Rev. Lett. **116**, 117602 (2016).

[8] B. W. Zingsem, M. Farle, R. L. Stamps, and R. E. Camley, "The unusual nature of confined modes in a chiral system," arXiv:1609.03417 (2016).
